# Deep learning‐based accurate diagnosis and quantitative evaluation of microvascular invasion in hepatocellular carcinoma on whole‐slide histopathology images

**DOI:** 10.1002/cam4.7104

**Published:** 2024-03-15

**Authors:** Xiuming Zhang, Xiaotian Yu, Wenjie Liang, Zhongliang Zhang, Shengxuming Zhang, Linjie Xu, Han Zhang, Zunlei Feng, Mingli Song, Jing Zhang, Shi Feng

**Affiliations:** ^1^ Department of Pathology, The First Affiliated Hospital, College of Medicine Zhejiang University Hangzhou P. R. China; ^2^ Department of Computer Science and Technology Zhejiang University Hangzhou P. R. China; ^3^ Department of Radiology, The First Affiliated Hospital, College of Medicine Zhejiang University Hangzhou P. R. China; ^4^ School of Management Hangzhou Dianzi University Hangzhou P. R. China

**Keywords:** deep learning, hepatocellular carcinoma, microvascular invasion, pathological diagnosis, whole‐slide image

## Abstract

**Background:**

Microvascular invasion (MVI) is an independent prognostic factor that is associated with early recurrence and poor survival after resection of hepatocellular carcinoma (HCC). However, the traditional pathology approach is relatively subjective, time‐consuming, and heterogeneous in the diagnosis of MVI. The aim of this study was to develop a deep‐learning model that could significantly improve the efficiency and accuracy of MVI diagnosis.

**Materials and Methods:**

We collected H&E‐stained slides from 753 patients with HCC at the First Affiliated Hospital of Zhejiang University. An external validation set with 358 patients was selected from The Cancer Genome Atlas database. The deep‐learning model was trained by simulating the method used by pathologists to diagnose MVI. Model performance was evaluated with accuracy, precision, recall, F1 score, and the area under the receiver operating characteristic curve.

**Results:**

We successfully developed a MVI artificial intelligence diagnostic model (MVI‐AIDM) which achieved an accuracy of 94.25% in the independent external validation set. The MVI positive detection rate of MVI‐AIDM was significantly higher than the results of pathologists. Visualization results demonstrated the recognition of micro MVIs that were difficult to differentiate by the traditional pathology. Additionally, the model provided automatic quantification of the number of cancer cells and spatial information regarding MVI.

**Conclusions:**

We developed a deep learning diagnostic model, which performed well and improved the efficiency and accuracy of MVI diagnosis. The model provided spatial information of MVI that was essential to accurately predict HCC recurrence after surgery.

## 1 INTRODUCTION

Hepatocellular carcinoma (HCC) is the sixth most common malignancy and the third leading cause of cancer‐related death worldwide.^1^ The prognosis for most patients with HCC is poor, with only an 18% 5‐year survival rate.^2^ Up to 80% of patients experience recurrence within 5 years after surgical resection,^2^ which is associated with microscopic foci of dissemination already present preoperatively. Microvascular invasion (MVI) refers to the presence of cancer cell nests microscopically seen in the portal vein, hepatic vein, or tumor envelope vessels within the paracancerous liver tissue.^3^ MVI is an independent prognostic factor that is associated with intrahepatic metastasis and early recurrence following the resection of HCC.^4^ HCC patients with MVI not only have a higher postoperative recurrence rate^5^ but also experience shorter recurrence time.^6^, ^7^ Effective postoperative adjuvant transarterial chemoembolization or sorafenib treatment for HCC patients with MVI significantly reduces tumor recurrence and improves survival.^8^, ^9^ Therefore, an accurate diagnosis of MVI is critical to the individualized treatment and follow‐up strategies for HCC.

Postoperative pathological assessment is generally considered as the “gold diagnostic standard” for MVI. However, the traditional pathology is relatively subjective, time‐consuming, and heterogeneous in the diagnosis of MVI. When multiple masses are present at once, counting MVI at each sampling site may become a time‐consuming and repetitive task. Furthermore, visual discrimination of micro MVI (i.e., less than 10 tumor cells) is challenging and even highly experienced pathologists may overlook or misdiagnose it. Detection rates of MVI vary widely between 7.8% and 74.4% in different pathologists.^10^ In addition, traditional pathology cannot provide precise information like the number of MVIs, tumor cell count or area of MVI, spatial information of MVI, and so on. The requirement for precise pathological diagnosis has challenged the traditional pathology.

The development of artificial intelligence (AI) in medicine has led to significant advancements in the diagnosis of tumors, assessment of drug efficacy, and prognosis prediction.^11^, ^12^, ^13^, ^14^, ^15^ In HCC, deep‐learning models based on histological whole‐slide images (WSIs) have been widely applied in diagnosis,^16^ pathological grading,^17^ molecular characterization^16^, ^17^ and prognostic assessment.^15^, ^18^ However, AI studies of MVI in HCC have mainly focused on preoperative prediction using radiomics,^19^, ^20^ which results in underestimation of the detection rate of MVI.^21^ As of now, there is no deep learning model that can rapidly and accurately assess postoperative histological MVI of HCC.

This study proposes the MVI artificial intelligence diagnostic model (MVI‐AIDM), which can significantly improve the efficiency and accuracy of MVI diagnosis leveraging our previously constructed framework for classifying blocks of HCC pathological images^22^ and microvascular segmentation.^23^ The proposed model simulates the MVI diagnosis method used by pathologists, involving three consecutive processes: detecting tumor regions, segmenting microvessels outside the tumor, and classifying cells in the microvascular. Exhaustive experiments have demonstrated the excellent performance of MVI‐AIDM in MVI diagnosis. Compared with pathologists, the proposed model not only improves the accuracy and efficiency of MVI diagnosis, but also provides quantitative and spatial information of MVI.

## 2 MATERIALS AND METHODS

### 2.1 Patient cohorts and ethics approval

Ethics approval for this study was obtained from the Ethics Committee of the First Affiliated Hospital, College of Medicine, Zhejiang University (FAHZJU). Written informed consent was obtained from all patients before surgery, and all personal information related to the patients was anonymized.

The study's patient selection involved a retrospective review of patients who underwent curative hepatectomy of primary HCC in FAHZJU from January 2018 to December 2021. We collected a total of 753 HCC patients from FAHZJU and 358 patients from The Cancer Genome Atlas (TCGA) database following the inclusion and exclusion criteria. All images and data from the TCGA are publicly available at https://portal.gdc.cancer.gov. The inclusion criteria consisted of patients who were (I) diagnosed with HCC after postoperative histopathology, (II) had a postoperative sampling in the First Affiliated Hospital, College of Medicine, Zhejiang University (FAHZJU) strictly following the 7‐point sampling protocol (SPSP),^24^ (III) did not undergo liver transplantation and (IV) had complete clinical and pathological data. Patients were excluded if they (I) had undergone any preoperative adjuvant treatment, (II) had a history of other malignancies, (III) were diagnosed with intrahepatic cholangiocarcinoma (ICC) or combined HCC–ICC postoperative histopathologically, (IV) had indistinct pathological images or obvious necrotic areas, and (V) had incomplete clinical and pathological data.

#### 2.1.1 Sampling protocol

**FIGURE 1:**
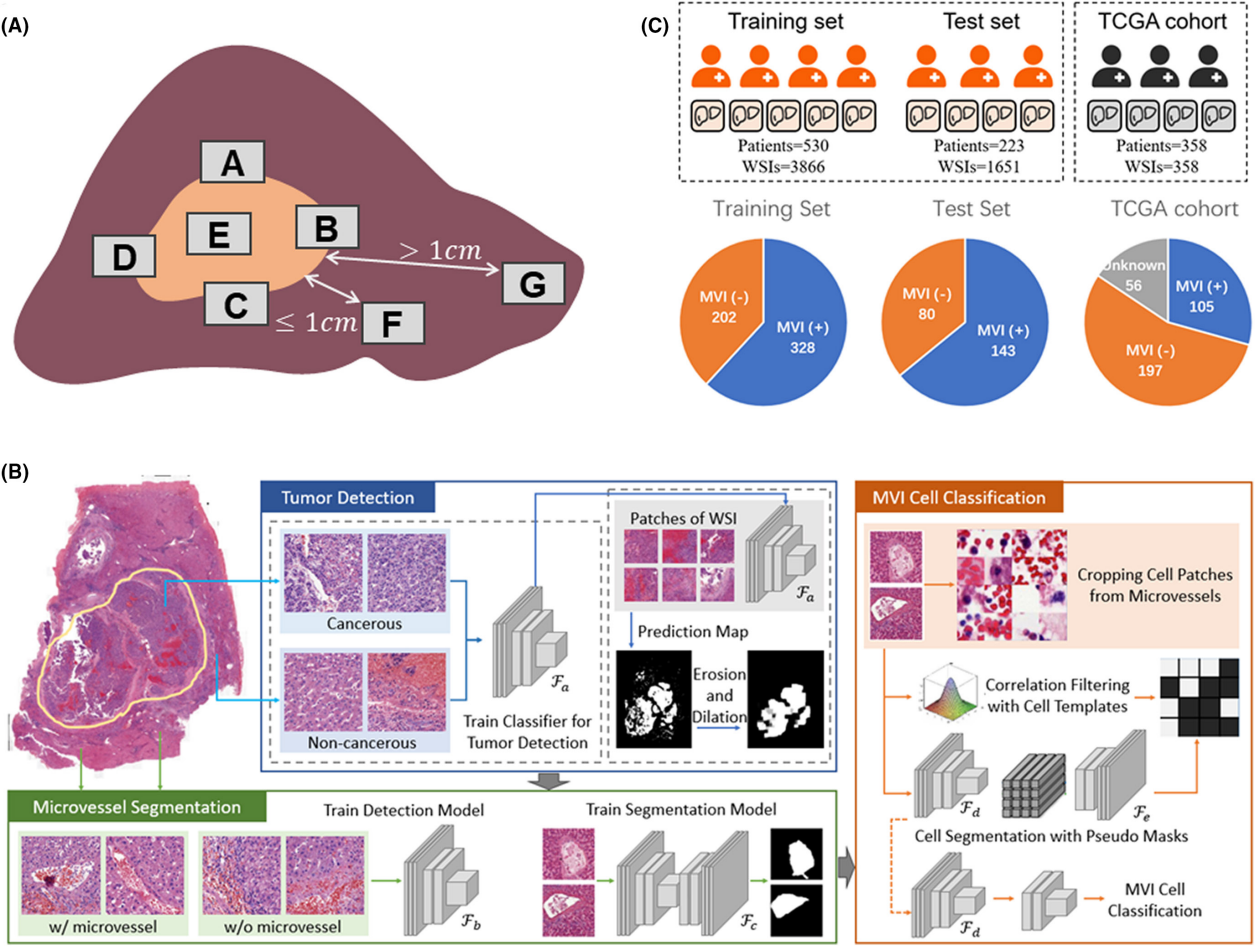
(A) Criteria of the seven‐point sampling protocol. (B) Illustration of the MVI diagnostic model. The proposed model consists of three processes including tumor detection, microvascular segmentation, and MVI cell classification. These processes are conducted in order, achieving the diagnosis of tumor region, cells and MVI. (C) The detail of dataset composition used in this paper. Our dataset includes 5517 WSIs from 753 patients, which is divided into training set and test set. In addition, TCGA cohort is applied for external validation. The amount of data with and without MVI is shown below.

Due to the high incidence of MVI located within 1 cm of the tumor margins,^25^ we performed postoperative sampling following the 7‐point sampling protocol (SPSP)^24^ as illustrated in Figure 1A. Tissue was harvested in a 1:1 ratio between the area of the tumor and that of the adjacent liver tissue at 4‐point positions (i.e., 12‐o'clock (A), 3‐o'clock (B), 6‐o'clock (C), and 9‐o'clock (D)) between the tumor and adjacent liver tissue. Additionally, tissue was harvested separately at the tumor center (E) and its periphery (≤1 cm away from the main tumor (F) and >1 cm away from the main tumor (G)). For multiple tumors, at least one block was obtained from each daughter nodule between the tumor and adjacent liver tissue, depending on the nodule size.

#### 2.1.2 Pathological diagnosis standard

Diagnosis of HCC was made using the Guidelines for the Diagnosis and Treatment of Hepatocellular Carcinoma (2019 Edition),^26^ while the diagnosis of MVI followed the practice guidelines for the pathological diagnosis of primary liver cancer.^24^ Two senior subspecialty pathologists identified HCC and MVI in all cases. In cases of disagreement, a third pathologist was involved in the identification process and provided the final result. The final diagnosis was defined as “pathologists' labels”.

### 2.2 Dataset preparation

A total of 5517 WSIs from 753 patients with HCC at FAHZJU were included in our study. The WSIs were randomly divided into a training set and a test set at a 7:3 ratio. All images were hematoxylin and eosin (H&E)‐stained and produced at 40× magnification using the 3DHISTECH P250 FLASH digital scanner (3DHISTEech, Budapest, Hungary). For validation purposes, we downloaded 358 slides of HCC from TCGA database as an external testing set. Since these slides only had case‐level labels, we manually annotated the presence of MVI on these slides for testing purposes.

### 2.3 Development of the deep learning model

We proposed a deep learning model for MVI diagnosis. As illustrated in Figure 1B, the model contains three processes, tumor region detection, microvascular segmentation, and MVI cell classification. The details of each process are provided in Data S1.

#### 2.3.1 Tumor region detection

The initial process of our proposed model involves detecting tumor regions. This process is trained using cancerous and noncancerous patches with rough annotations. To handle the issue of noisy labels, we propose a pathological classification framework that utilizes a noise‐rectifying (NR) loss function. Predictions are made on individual patches, and these predictions are combined to create the prediction maps of WSIs. These maps are then post‐processed using erosion and dilation operations in order to obtain the main tumor region.

#### 2.3.2 Microvascular segmentation

Once the main region of the tumor has been detected, the next step of our model is to locate and segment the microvessels outside of the tumor. To accomplish this, we employ the ResNet18 classification model, which is trained to detect patches containing microvessels. ResNet uses skip connections to address the vanishing gradient problem, which helps achieving successful training for deep classification networks. This model is trained on both microvascular‐containing and microvascular‐free patches. Additionally, we use the DeepLabv3 model for semantic segmentation in pathological patches with microvessels. DeepLabV3 is a state‐of‐the‐art deep learning model for semantic image segmentation. It has precise object recognition and boundary detection ability and can achieve great performance in microvascular segmentation.

#### 2.3.3 Microvascular invasion cell classification

After obtaining patches from the microvessels outside the tumor region, the critical step is to classify the cells and diagnose MVI. To train models without cell annotations, we propose a weakly supervised method for cell classification using only patch‐level labels. Our proposed model consists of a cell localization branch and a cell classification branch with the encoder that shares parameters. By incorporating mutual feedback between two branches, the encoder is able to achieve better performance in both locating and diagnosing the cells within the microvessels.

### 2.4 Microvascular invasion reviews by 6 pathologists for comparsion

To validate the performance of the MVI‐AIDM model at the WSI level, we invited six subspecialty experienced pathologists (three senior pathologists and three intermediate pathologists) to independently assess WSIs in the FAHZJU test set by traditional pathology. As described above, the final diagnosis by three senior pathologists was defined as “pathologists' labels”. We compared the detection rate and time cost of diagnosing MVI between pathologists and MVI‐AIDM.

### 2.5 Spatial information evaluation

In this study, we evaluated the performance of our model in capturing spatial information using several metrics, including the distance from the MVI to the tumor, the area ratio of the MVI, and cancer cells counting. By analyzing the results from the three processes, we were able to calculate the distance between the MVI and the tumor. Additionally, the area ratio of MVI and cancer cells amount can be calculated by using the segmentation and correlation filtering results. Overall, these metrics offer valuable information regarding the spatial relationship between the MVI and tumor region, providing important insights for diagnostic and treatment decision‐making.

### 2.6 Statistical analysis

We evaluated the performance of our proposed model using several metrics, including accuracy, precision, recall, F1 score, the receiver operating characteristic (ROC) curve, and the area under the curve (AUC) for classification. In the segmentation task of microvessels, we used MPA, MIoU, FWIoU, and DICE as widely applied metrics for evaluation. These metrics were used to assess the accuracy and effectiveness of the model in segmenting microvessels.

In order to validate the performance of the MVI‐AIDM model at the WSI level, we compared the positive rate of MVI between our proposed model and six subspecialty experienced pathologists. We utilized the Mc‐Nemar test to compare the positive rates of MVI. The *p* < 0.05 is considered statistically significant. By using this method, we were able to validate the accuracy of our model on detecting MVI and compare its performance to that of pathologists.

## 3 RESULTS

### 3.1 Baseline features of dataset

Our study included a total of 5517 slides from 753 patients diagnosed with HCC at FAHZJU. Of these patients, 471 (62.55%) were confirmed to be positive for MVI. To develop and validate our proposed model, patients from the FAHZJU cohort were randomly divided into a training set and a test set at a 7:3 ratio, consisting of 3866 slides from 530 patients in the training set and 1651 slides from 223 patients in the test set. The positive rate of MVI for these sets was 61.89% and 64.13%, respectively. For validation purposes, we also utilized an external test set from the TCGA database, which included 358 slides from 358 HCC patients (as shown in Figure 1C). Baseline clinicopathological and demographic characteristics were generally well‐balanced between the FAHZJU and TCGA cohorts, as indicated in Table 1. Additionally, we analyzed the size distribution of the WSIs and found them to have varying widths and heights, as illustrated in Figure S1.

**TABLE 1:** Baseline clinicopathological and demographic characteristics of HCC patients.

Variable	Value	Training set		Internal test set		External test set	
		*N*	%	*N*	%	*N*	%
Total patients		530		223		358	
Age (years)	<60	275	51.89	112	50.22	164	45.81
	≥60	255	48.11	111	49.78	193	53.91
	Unknown	0	0	0	0	1	0.28
Gender	Female	89	16.79	32	14.35	116	32.40
	Male	441	83.21	191	85.65	242	67.60
AFP level	Normal	197	37.17	75	33.63	141	39.39
	Abnormal	330	62.26	146	65.47	129	36.03
	Unknown	3	0.57	2	0.90	88	24.58
Tumor number	Single	449	84.72	189	84.75	330	92.18
	Multiple	81	15.28	34	15.25	26	7.26
	Unknown	0	0.00	0	0.00	2	0.56
Tumor diameter (cm)	≤3	238	44.91	118	52.91	74	20.67
	>3	292	55.09	105	47.09	282	78.77
	Unknown	0	0.00	0	0.00	2	0.56
Tumor differentiation	I	17	3.21	14	6.28	52	14.53
	II	277	52.26	115	51.57	170	47.49
	III	207	39.06	87	39.01	121	33.79
	IV	29	5.47	7	3.14	11	3.07
	Unknown	0	0.00	0	0.00	4	1.12
AJCC stage	I	303	57.17	129	57.85	166	46.37
	II	184	34.72	73	32.73	82	22.90
	III	38	7.17	19	8.52	84	23.46
	IV	5	0.94	2	0.90	4	1.12
	Unknown	0	0.00	0	0.00	22	6.15
MVI	Yes	328	61.89	143	64.13	105	29.33
	No	202	38.11	80	35.87	253	70.67

Abbreviations: AFP, alpha‐fetoprotein; AJCC, American Joint Committee on Cancer; HCC, hepatocellular carcinoma; MVI, microvascular invasion.

### 3.2 Tumor classification and region visualization

**FIGURE 2:**
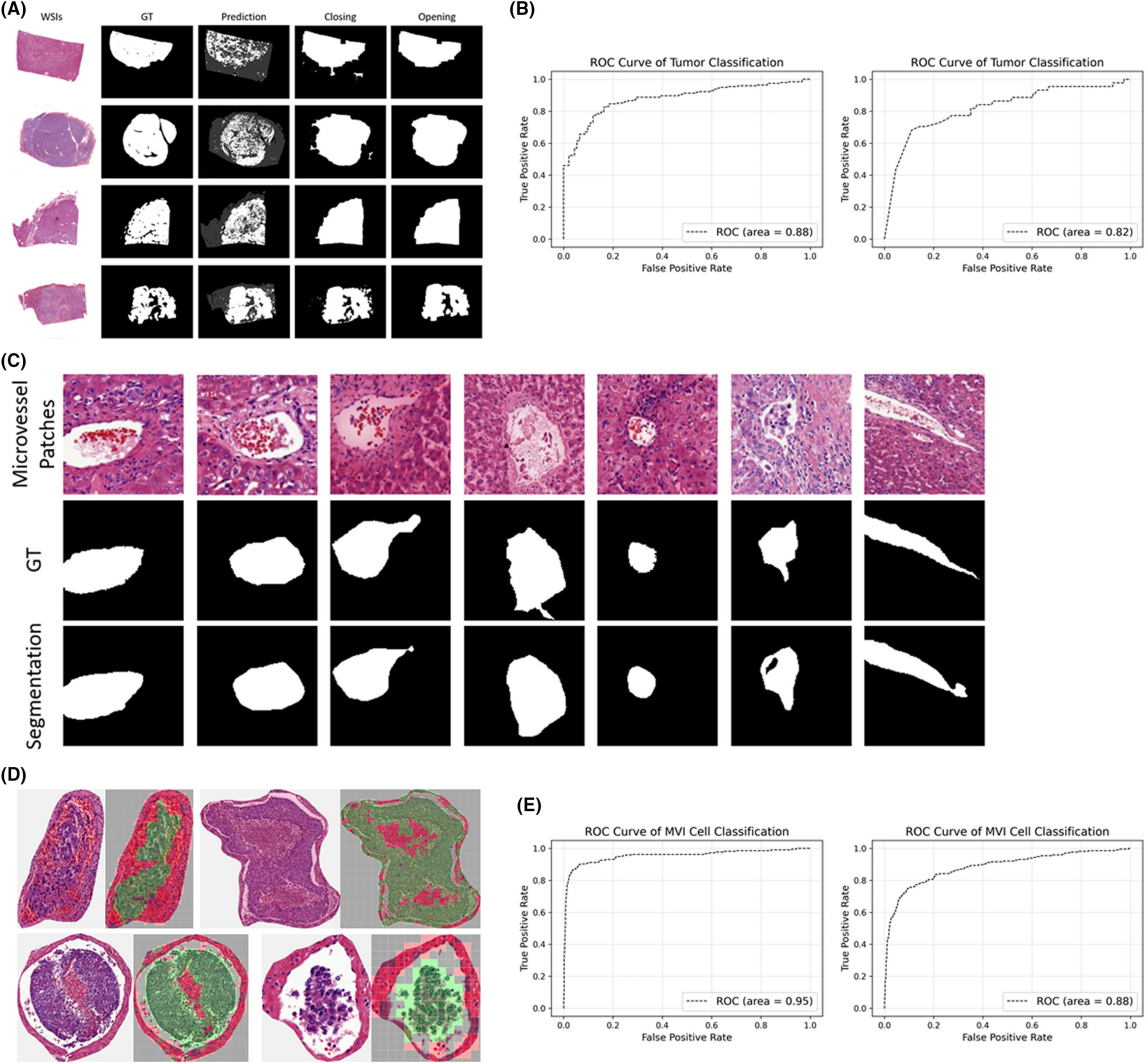
Experimental results of three processes of our model. (A) Results of the tumor region detection. GT denotes the ground truth annotated by pathologists. The third column shows the predicted maps. The last two columns show the results of conducting closing operation and opening operation, which generating the final tumor region. (B) The ROC of tumor classification of our dataset (left) and TCGA dataset (right), achieving the AUC of 0.88 and 0.82. (C) Results of the microvascular segmentation on HCC pathological images. The first line is the cropped microvascular patches. The second line of GT is the ground truth annotated by pathologists. The third line is the segmentation results. (D) Visualization of the MVI diagnosis results. The microvascular samples are cropped into patches and classified. From the visualized results, the red patches denote the regions with normal cells, and the green patches denote the regions with cancer cells. Cells with green points are cancer cells annotated by pathologists. (E) The ROC of MVI cell classification of our dataset (left) and TCGA dataset (right), achieving the AUC of 0.95 and 0.88.

During the evaluation stage, the WSIs are cropped into small patches that serve as inputs to the well‐trained classification model, resulting in the production of prediction maps. To isolate the target tumor region, the post‐processing based on erosion and dilation operations is performed. Figure 2A illustrates annotated tumor regions, the prediction maps, and the post‐processing results. From the comparison of annotations and prediction maps, the latter contain scattered regions. Initially, dilation and erosion operations are conducted successively on original prediction maps to fill the negative spaces inside the predicted tumor region. Then erosion and dilation operations are conducted to filter the positive areas outside the tumor. The visualization results show that the predicted tumor regions are more complete after the post‐processing compared to the original ones. Our model achieves an AUC value of 0.88 and 0.82 in the FAHZJU test set and TCGA cohort, respectively (Figure 2B). The accuracy of tumor region detection exceeds 0.92 in the FAHZJU test set (Table S1). And the post‐processing strategies lead to accurate tumor region detection for the following processes.

### 3.3 Microvascular segmentation results

After detecting the tumor in each WSI, we isolate the corresponding region and proceed to perform microvascular detection and segmentation on the remaining parts of the WSI. Next, we screen potential regions for suspicious areas, including annular regions with ample space and filled with cancer cells. Subsequently, pathologists perform sample screening on the candidates, which may include little false positive MVIs, and we conduct microvascular segmentation. In microvascular detection, the detected patches are combined through post‐processing for segmentation (Figure S2). During the segmentation model's evaluation, representative microvascular samples are shown in Figure 2C, including the first four with significant blank space, followed by two patches with relatively small sizes, and finally, one with a narrow shape. These samples are accurately annotated by pathologists, indicated as “GT.” From a comparison of these annotations and the segmentation outcomes, it can be observed that the predicted segmentation results closely match the annotated ones. Even for samples with small targets, our model can accurately segment the microvessels.

Additionally, we present quantitative results in Table S2 using various metrics for segmentation, including MPA, MIoU, FWIoU, and DICE. The model's performance is evaluated on normal patches and MVI patches separately. The performance on normal patches is superior, due to the more complex nature of the MVI samples than the normal ones. But the visualization results show that our model can achieve superior segmentation results for both normal and MVI microvessels.

### 3.4 Microvascular invasion cell classification results

The cell classification results in MVI are shown in Figure 2D. Patches with negative predictions are visualized in red, and patches with positive predictions are visualized in green. The nucleus of cancer cells are annotated with green points. From the overall effect of visualization, our model effectively detects all the cells in each patch (red and green predictions). In addition, almost all the green points are covered with green patches, which demonstrates that our model has the good performance on detecting cancer cells in MVI, with an AUC value of 0.95 and 0.88 in FAHZJU test set and TCGA cohort, respectively (Figure 2E).

Besides, quantitative experiments of MVI cell classification are conducted and the results are shown in Table S3. The evaluation is conducted based on pathologist's annotations with metrics including precision, recall, and F1 score. It can be seen that the recall is higher than precision in cell locating, which represents our model tends to detect cancer cells. In clinical diagnosis of MVI, it is crucial to detect as many cancer cells as we can. The high recall of our model will miss very few cancer cells, which effectively assists pathologists to assess the severity of the spread.

### 3.5 Performance of MVI‐AIDM model

For the final MVI diagnosis, our model classifies the entire microvessels as normal or MVI based on the predictions of the three processes discussed above. As shown in Table 2, our model achieves high accuracy at the patch level in both the internal and external test sets, with accuracy values of 97.49% and 94.25%, respectively. This excellent performance indicates that our model can effectively diagnose MVIs. Additionally, we calculate the precision and recall to evaluate the model's performance on normal and MVI vessels, respectively. The results show that normal vessels are better identified than MVI, likely because of the simple and uniform features of normal patches. Nevertheless, with a precision of 92.44% and a recall of 92.98% in the FAHZJU test set, our model still has a powerful ability to classify most MVIs from all the microvessels in each WSI.

**TABLE 2:** The performance of MVI‐AIDM on the patch level.

MVI Diagnosis	Accuracy	Precision		Recall	
		Normal	MVI	Normal	MVI
Internal test set	0.9749	0.9855	0.9244	0.9843	0.9298
External test set	0.9425	0.9487	0.9011	0.9539	0.9076

Abbreviations: MVI, microvascular invasion: MVI‐AIDM, MVI artificial intelligence diagnostic model.

### 3.6 Comparison of MVI‐AIDM model to pathologists

**FIGURE 3:**
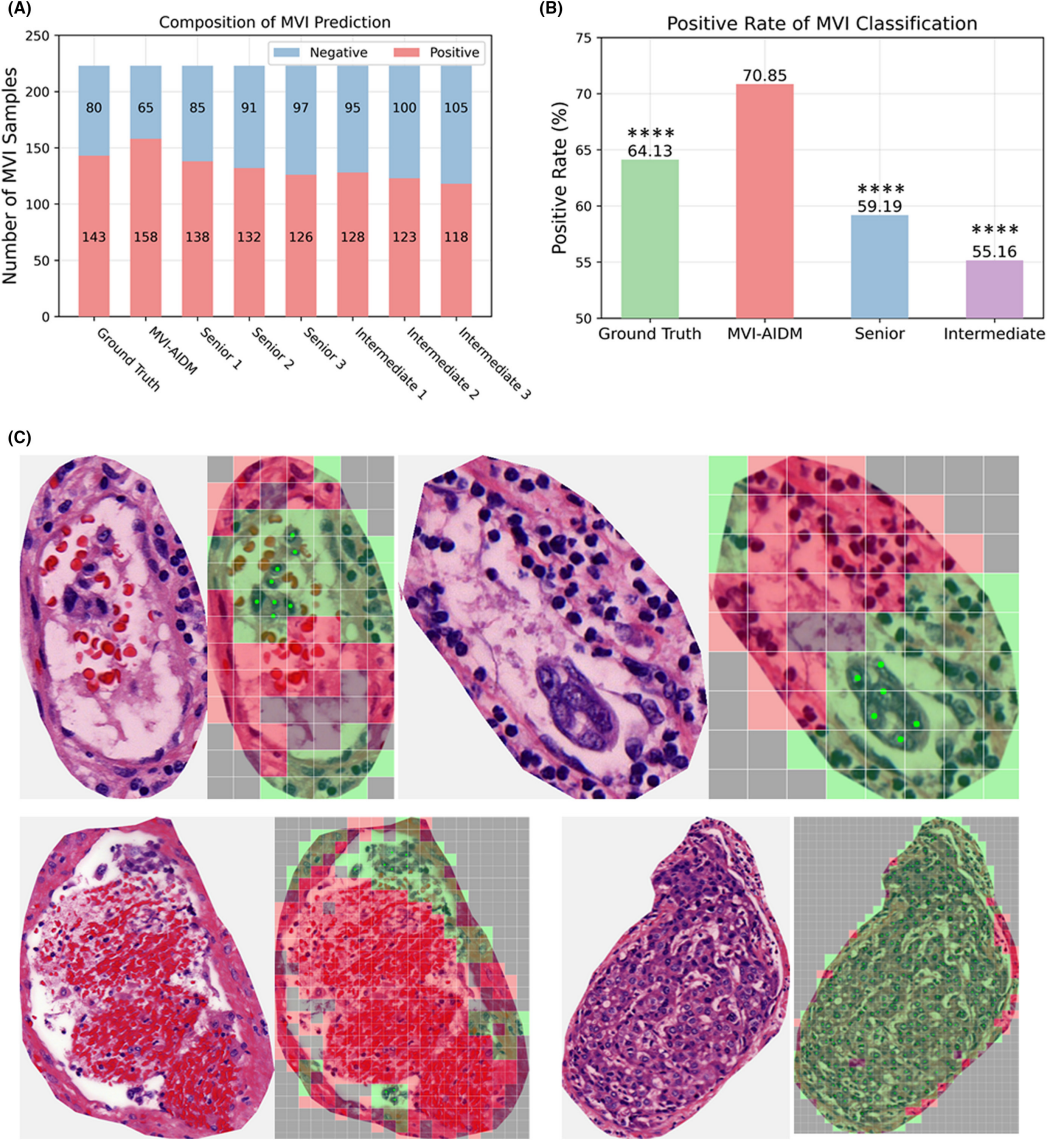
(A) The composition results of MVI prediction for our proposed MVI‐AIDM and six pathologists. The number of MVI is given to shown the proportion of negative (blue) and positive (red) samples. (B) The comparison for the positive rate of MVI classification. The “Senior” and “Intermediate” denote the average results of senior and intermediate pathologists. ****p < 0.0001 (C) The reasons for missed diagnosis of MVI by pathologists were: micro MVI (<10 tumor cells, the left top two images), interference from inflammatory cells (the right top two images) and interference from erythrocytes (the left bottom two images), challenges in identifying micro satellite nodules (the right bottom two images). The red patches and the green patches denote the regions with normal cells and cancer cells, individually. Cells with green points are cancer cells annotated by pathologists.

As illustrated in Figure 3A,B, the MVI positivity rates diagnosed by the six subspecialty pathologists ranged from 52.91% to 61.88%. By comparison, the MVI‐AIDM model diagnosed a total of 158 positive cases of MVI from 223 cases in the test set. Consequently, the positive rate of MVI was significantly increased to 70.85% (*p* < 0.001), even beyond that of the “pathologists labels” (64.13%, *p* < 0.001).

We noticed that 15 cases were diagnosed as MVI negative by the “pathologists labels” but positive by the model. Immunohistochemistry (IHC, i.e., CD31, CD34, Hepatocyte, and GPC‐3) was performed to aid the interpretation of difficult diagnostic cases of MVI. We provided the corresponding patches of these 15 cases' WSIs to 6 subspecialty pathologists for analysis in a human‐machine interactive manner. Assisted by the model and IHC, 13 of these cases were re‐diagnosed as MVI positive. The reasons for these false negatives are presented in Table S4. Further, visual analysis revealed the causes of false negatives, including micro MVI (<10 tumor cells), interference from inflammatory cells and erythrocytes, and challenges in identifying micro satellite nodules (Figure 3C). In two cases, contamination at the time of sampling might have caused the errors.

In conclusion, the adoption of the MVI‐AIDM model significantly improves the detection rate of MVI as an auxiliary diagnostic tool. Additionally, this model greatly shortens the manual diagnosis procedure of MVI. In the test set, it took a senior pathologist 28.7 ± 11.9 min to accurately assess MVI in a case. In contrast, MVI‐AIDM took only 9.1 ± 4.9 min from slide scanning to result output, significantly enhancing the efficiency of MVI diagnosis (*p* < 0.01).

### 3.7 Evaluation of the spatial information presented in microvascular invasion

**FIGURE 4:**
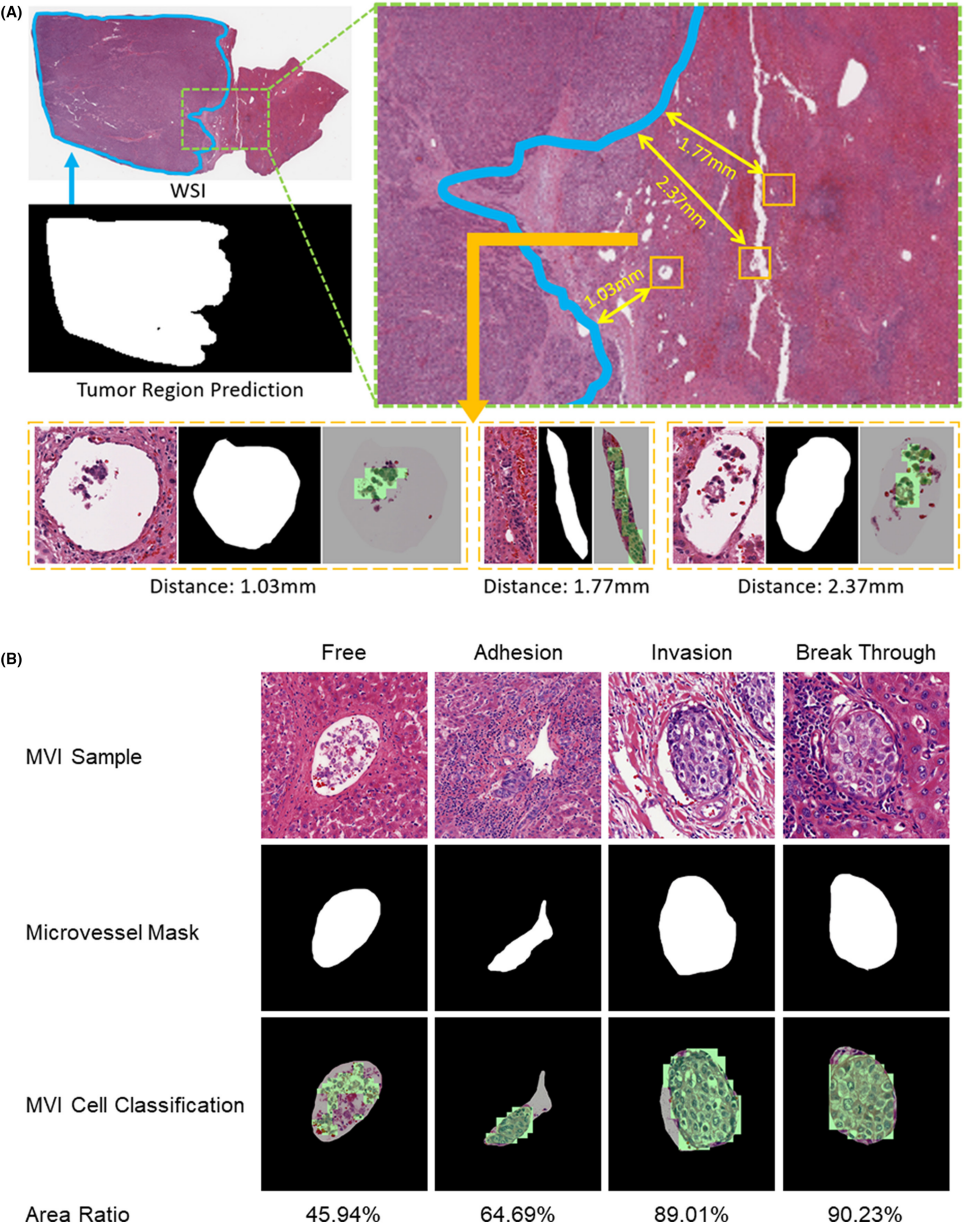
(A) Visualization of the spatial information of MVI. Based on the results of three processes, the position of tumor region (blue) and MVIs (yellow) can be determined. The corresponding distance can be calculated. (B) Visualization of relationship between MVI and microvascular walls, including free, adhesion, invasion, and break through. Based on the prediction of microvascular and cancer cells, the area ratios can be calculated, which are shown below.

MVI‐AIDM has the potential to quantify subvisual information accurately compared to manual diagnosis. For instance, our model can calculate the spatial information of MVI by using its novel 3‐step process, which includes determining the distance between MVI and tumor, the number of cancer cells in MVI, and the area ratio. Figure 4A provides an example illustrating the calculation of the spatial information based on the results of the three processes. Initially, the tumor prediction results are used to determine the extent of the tumor region. Next, the model detects and recognizes all the MVIs in the predicted microvascular locations. Finally, the distance between each MVI and the tumor is measured.

In addition, Figure 4B presents examples of MVIs and their varying relationships with tumor cells and microvascular walls. The four scenarios include: (I) free MVI (nonadherent to the endothelium), (II) adhesion MVI (adhered to the endothelium), (III) invasion MVI (adhered and invading the endothelium), and (IV) breakthrough MVI (penetrated the microvascular wall). Based on the results of microvascular segmentation and MVI cell classification, our model can directly calculate the microvascular area and cancer cells area. Additionally, the area ratio of cancer cells to the microvascular area can be obtained, which reflects the degree of invasion. Moreover, MVI cell counting is a crucial metric in the MVI diagnosis, and it is achieved through applying correlation filtering results.

## 4 DISCUSSION

Several retrospective studies have demonstrated the critical role of MVI as a determinant of early recurrence in HCC patients after surgical resection and liver transplantation.^27^, ^28^, ^29^ In a study conducted by Lim et al.,^27^ the prognosis of 454 patients who underwent radical surgical resection was evaluated, and it was observed that MVI was a more accurate predictor of recurrence and survival outcomes than Milan criteria. The precise pathological identification of MVI is essential for treatment decisions in HCC patients. However, histopathology, the “gold standard” for MVI diagnosis, has limitations in accurately and rapidly identifying MVI and assessing its spatial information. In this study, we developed the MVI‐AIDM model to evaluate MVI in HCC patients. Our model was subsequently validated using an independent external cohort and demonstrated remarkable accuracy in identifying MVI and quantifying some of its essential spatial information.

In this study, the deep learning model was developed for automatic quantitative assessment of MVI by simulating the diagnostic approach of a pathologist, using a 3‐step approach: tumor region localization, microvascular segmentation, and cell classification. Our study demonstrated that MVI‐AIDM can accurately and rapidly identify MVI in HCC at different stages and grades. At the patch level, MVI‐AIDM achieved a high accuracy rate of 94.25% in the independent external validation set. Interestingly, our results revealed that MVI‐AIDM had a higher MVI positive detection rate than traditional microscopic diagnosis (70.85% vs. 64.13%, *p* < 0.001). This implies that MVI‐AIDM surpasses traditional pathology with its performance, and can assist pathologists in identifying micro MVI and some MVIs that are difficult to identify controversially.

In recent years, few deep learning studies have been conducted on MVI detection through WSI. Most of them were retrospective with small sample sizes. Yu et al.^30^ developed a macroscopic histological slide that covered the entire cut surface of a surgical specimen, which improved the detection rate of MVI by matching digital macro‐slides in a cohort of 91 HCC patients. However, the method does not automatically identify MVI from a macro‐slide, and it requires large‐scale manual annotation. This process can be time‐consuming, laborious, and easily overlooks micro MVIs (<10 tumor cells). Sun et al.^31^ implemented a PCformer model to identify MVI regions by classifying MVI boundaries according to 69 HCC‐WSIs. However, the model's F1‐score was only 80.06%, and independent validation was not conducted. Additionally, differentiating MVI from satellite nodules can be challenging. Chen et al.^32^ developed a deep learning model that predicted MVI from tumor areas of histology images in a cohort of 350 HCC patients. The model achieved an AUC of 0.871 and had predictive significance for HCC patients with narrow surgical margins (<0.5 cm) or biopsy only. However, 83.3% of the MVIs were located within 1 cm from the tumor boundary.^24^ Wide margin resection can effectively reduce tumor recurrence and improve survival of HCC patients with MVI.^33^ For a vast majority of HCC patients, full postoperative sampling is needed for evaluation of MVI. In this paper, our novel approach to automated MVI detection mimicked pathologists' method by identifying vessels and tumor cells. Our MVI‐AIDM model can precisely identify cancerous or noncancerous regions, segment microvessels, and accurately identify tumor cells in microvessels. Additionally, this model enables quick, accurate MVI count, and can calculate tumor cellularity (accurate to single digits) and MVI area. Furthermore, it can quantify MVI spatial information (distance from the tumor or operative margin) and the relationship between MVI and microvascular, providing important subvisual information. To our knowledge, our study is the first to automatically quantify both the tumor cellularity and spatial information of MVI in pathology images. With the development of digital pathology, our model can serve as an auxiliary tool for pathologists to diagnose MVI, and has the potential for clinical transformation in the future.

Training a model to handle rough annotations in pathological images requires a robust strategy to address the impact of noisy labels. Prior research in noisy‐label learning has focused on model‐based and model‐free methods. Model‐based strategies try to differentiate between clean and noisy samples using sample screening techniques,^34^ whereas model‐free approaches incorporate anti‐noise loss functions into commonly used models.^35^ However, in the context of pathological images, mislabeled patches are specifically related to the surrounding information. As a result, our proposed model accounts for the distinct features of pathological images, providing critical information for tumor detection.

Even though there are numerous fully‐supervised methods for cell detection^36^, ^37^ and segmentation^38^, ^39^ available, their performance relies heavily on a significant number of accurately annotated data. Weakly supervised approaches have been explored by Mahmood et al.^40^ and Feng et al.^41^ using synthetic samples, original annotated samples, and a mutual‐complementing model of optimized detection and segmentation branches. However, due to the unique features of MVI samples in pathological images, these methods cannot directly detect and segment cancer cells without adequate cell‐level annotations. Our proposed MVI classification model employs correlation filtering, enabling it to carry out accurate cell segmentation without any cell annotation. The two branches of the proposed model take full advantage of MVI sample characteristics and result in excellent performance in both cell segmentation and MVI classification.

There are a few limitations in our study. First, MVI is a significant prognostic factor for HCC. However, there is still considerable international debate regarding MVI grading. Multiple research teams have analyzed the MVI patterns associated with prognosis to propose novel staging criteria. Sumie et al.^42^ evaluated 207 surgically resected HCC samples, with a particular emphasis on the burden of MVI amount. Iguchi et al.^43^ evaluated 142 patients treated with liver transplant for HCC, believing that the tumor cellularity of MVI (>50 cells) was predictive of recurrence‐free survival. Feng et al.^44^ put particular emphasis on the impact on prognosis of the relationships between MVI and microvascular. We will further study the grading standard of MVI and factors associated with prognosis via MVI features extracted by the model. Second, there are three types of MVIs in HCC: portal invasion, hepatic vein invasion, and hepatic artery invasion. We will attempt to recognize three types of MVI by MVI‐AIDM in future research. Third, our model may exhibit little false positives owing artifactual displacement of cells into microvascular lumen during processing that require further verification by pathologists. In this manuscript, we provide adequate training samples for the three tasks. Insufficient samples often result in model overfitting in deep learning. In future research, we will investigate the impact of varying sample sizes on the diagnostic performance of the model.

## 5 CONCLUSIONS

In conclusion, we developed an AI diagnostic model for the rapid and precise identification of MVIs in WSIs. The model can evaluate and visualize the information embedded in MVI concurrently. This accomplishment can aid pathologists in MVI diagnosis, enhance the detection rate of MVIs, and provide additional groundwork for the standard grading of MVI. Clinicians can use this model to assess recurrence risk and formulate the best individualized therapeutic or management strategies for HCC patients. There is a wide potential for the clinical application of this project.

## AUTHOR CONTRIBUTIONS

**Xiuming Zhang:** Resources (equal); writing – original draft (equal). **Xiaotian Yu:** Investigation (equal); software (equal). **Wenjie Liang:** Data curation (equal); resources (equal). **Zhongliang Zhang:** Software (equal); validation (equal). **Shengxuming Zhang:** Software (equal); validation (equal). **Linjie Xu:** Resources (equal). **Han Zhang:** Resources (equal). **Zunlei Feng:** Software (equal); supervision (equal). **Mingli Song:** Software (equal); validation (equal). **Jing Zhang:** Funding acquisition (equal); project administration (equal). **Shi Feng:** Data curation (equal); validation (equal).

## FUNDING INFORMATION

This study was supported by grants from the Natural Science Foundation of Zhejiang Province (LY21H160035, LQ20H160048), the National Natural Science Foundation of China (62002318, 81971686, 62376248).

## CONFLICT OF INTEREST STATEMENT

The authors declare no potential conflicts of interest.

## ETHICS STATEMENT

Ethics approval for this study was obtained from the Ethics Committee of the First Affiliated Hospital, College of Medicine, Zhejiang University. Written informed consent was obtained from all patients before surgery, and all personal information related to the patients was anonymized. This study was performed in accordance with the ethics standards of the participating institutions and the tenets of the Declaration of Helsinki.

## CONSENT FOR PUBLICATION

All authors confirm their consent for publication the manuscript.

## Supporting information


Data S1.

## Data Availability

To protect the privacy of the patients, the data are not available for public access. However, the data are available from the First Affiliated Hospital, College of Medicine, Zhejiang University for researchers who meet the criteria for access to confidential data. Interested researchers can send data access requests to the corresponding author (fengshi@zju.edu.cn) upon reasonable request. TCGA data are available online approving by the Ethics Committee.
