# Supplementary material for: Deep learning‐based accurate diagnosis and quantitative evaluation of microvascular invasion in hepatocellular carcinoma on whole‐slide histopathology images
Source: Cancer Med. 2024 Mar 15;13(5):e7104. doi: 10.1002/cam4.7104 (PMC10941532; doi:10.1002/cam4.7104)
Supplement: Supplementary file 1 — Data S1. [file CAM4-13-e7104-s001.docx]

**Supplementary methods**

**Tumor Region Detection**

The first process of our proposed framework is tumor region detection. Since the subsequent microvascular segmentation and microvascular invasion (MVI) diagnosis should be conducted outside the tumor, this process facilitates the identification of region for following tasks. In this process, our classification model applies ResNet18^1^ as the backbone, which is pre-trained on ImageNet.^2^ As mentioned in dataset preparation, this model is trained with cancerous and non-cancerous patches. These patches are labeled based on the tumor annotations. However, due to the large size of pathological image, the annotations are inevitably rough, which introduces the noisy-label problem. It means the annotated labels of some patches are wrong. During model training, these noisy labels can mislead the model and cause poor classification performance.

Aiming at this problem, we propose the pathological classification framework with noise-rectifying (NR) loss function, which is defined as follows:

$\mathcal{L}_{NR}=-\log\left( 1-p^{\alpha}\left( 1-p \right)^{1-\alpha} \right)$,

where $p$ denotes the binary prediction of each sample being cancerous, $\alpha$ denotes the noise coefficient than ranges from 0 to 1. The parameter $\alpha$ controls the tendency of the model training. With a small value of$\alpha$, the model training would tend to the annotated labels. When $\alpha=0$, the proposed NR loss equals to $-\log p$, which is exactly the regular cross-entropy (CE) loss. Conversely, when the annotated label is highly unreliable, a large value of $\alpha$ is adopted to reverse the model tendency of this label. In our framework, the $\alpha$ is determined dynamically according to the surrounding feature. The detailed theoretical analysis is given in our previous research.^3^ Eventually, through our proposed framework with NR loss, the model can be trained robustly even if the tumor regions are roughly annotated.

With the well-trained model for pathological classification, we cropped each WSI into patches and predict individually. Then the results of all patches are combined based on the corresponding positions, generating the prediction map. However, since the model predictions are conducted based on the patch-level, there are some scattered regions whose results are inconsistent with the surroundings. For example, patches with MVI will be classified as positive, and some patches inside the tumor do not contain cancerous feature and will be classified as negative (such as red blood cells).

Considering the purpose of detecting tumor region is to identify microvessels outside the tumor, we need to obtain the complete tumor region through post-processing. This paper proposes the strategy base on erosion and dilation operations. Through specific kernels, erosion operation compresses the target region and expands the surrounding background, while dilation operation is just the opposite. Combining these operations in different orders can achieve different effects. To obtain the tumor region, erosion-dilation is firstly operated in order on the predicted tumor region, removing the scattered cancer areas outside the main region. Then inversely, dilation-erosion is operated to fill the internal area of tumor. Through the above processes, scattered areas inside and outside the tumor will be rectified, so that the main tumor region can be determined.

The hyper-parameters used for tumor region detection are set as follows. In the training stage, the batch size is set to 128 and the initial learning rate is set to 0.01. The learning rate is decayed in a cosine annealing manner. The SGD is adopted as the optimizer, with the momentum of 0.9 and weight decay of 0.0001. The training epoch is set to 20. The kernel size of erosion and dilation is set to 6 for removing scattered cancer areas, and the kernel size of dilation and erosion is set to 16 for closing the space within the tumor. For the dataset, 165510 patches are randomly cropped from the foreground of all 5517 slides. According to the annotated tumor region, there are 79183 cancerous patches and 86327 non-cancerous patches for binary classification of the first process. With $50\times$ magnification, these patches are all cropped with size 448 and resize to 224.

**Microvascular Segmentation**

After detecting the main region of tumors, the next process is to locate and segment the microvessels outside the tumor. A total of 28950 patches with microvessels are cropped in this stage, and the microvessels are annotated in these patches. In addition, same amount of patches without microvessels are randomly cropped outside the tumor region. These patches with binary labels are used in this process to train the classification model for detecting microvessels, and the patches with microvessels are used to train the model for microvascular segmentation and MVI classification. All these patches are cropped and resized to 224. The well-trained classifier $F_{b}$ is used to search microvessels from the whole-slide images of the testing set, and the segmentation model $F_{c}$ is used to segment the microvessels for the detected patches.

Before segmentation, the first step is to locate all microvessels in the whole-slide images. ResNet18 is adopted as the classification model $F_{b}$ to detect whether a patch contains microvessels. In the training stage, patches and the corresponding binary labels (with and without microvessels) are divided as the training set and validation set with the ratio of 10:1.

In this study, the semantic segmentation model DeepLabv3^4^ is adopted to segment microvessels in pathological patches. All patches with microvessels and a few patches without microvessels are used in the training stage. The annotations of the former are used as targets, where the value of 1 represents the microvascular region and the value of 0 represents the background. The targets of the latter are all set to 0. After training, the segmentation model $F_{c}$ is used to segment the region of vessels, making it convenient to diagnosis MVI in the microvessels.

This section contains two models, detection model $F_{b}$ and segmentation model $F_{c}$. Both models use SGD as the optimizer, and the momentum and the weight decay are set to 0.9 and 0.0001, respectively. For $F_{b}$, the batch size is set to 128, the initial learning rate is set to 0.01, and the training epoch is set to 100. And for Fc, the batch size is set to 64, the initial learning rate is set to 0.0001, and the training epoch is set to 300.

**MVI Cell Classification**

The crucial task of MVI cell classification is conducted after obtaining patches from the microvessels outside the tumor region. Initially, individual cell patches are cropped from each microvascular. Since there is no existing annotation for cell localization, we use the correlation filtering method^5^ to locate cells. Various cells are chosen at random to serve as templates for generating pseudo masks, which are then used as targets of the inputted cell patches for localization. Our proposed model in this process utilizes ResNet-50^6^ as the backbone for the shared encoder $F_{d}$, which is used in both the cell locating and classification branches. In the cell locating branch, we apply a deconvolution network, $F_{e}$, to output cell locating predictions. This branch is trained with the supervision of pseudo masks and is primarily designed to enable the encoder $F_{d}$ to learn cellular features more effectively. In the cell classification branch, the shared encoder is connected to a classifier that outputs predictions for each cell patch. These predictions are then aggregated for each microvascular, producing the final MVI diagnosis results. Microvessels containing cancerous cells are visualized in the WSI to facilitate interpretation and diagnostic decision-making.

**Reference**

1. Zhou KQ, Sun YF, Cheng JW, et al. Effect of surgical margin on recurrence based on preoperative circulating tumor cell status in hepatocellular carcinoma. EBioMedicine 2020, 62:103107. https://doi.org/10.1016/j.ebiom.2020.103107

2. Deng J, Dong W, Socher R, Li LJ, Li K, Li F. Imagenet: A large-scale hierarchical image database[C]//2009 IEEE conference on computer vision and pattern recognition. Ieee, 2009: 248-255.

3. Yu X, Feng Z, Song M, Wang Y, Zhang X, Li T. Tendentious Noise-rectifying Framework for Pathological HCC Grading[C]//British Machine Vision Conference. 2021.

4. Chen LC, Papandreou G, Schroff F, Adam H. “Rethinking atrous convolution for semantic image segmentation, in Conference on computer vision and pattern recognition (CVPR). IEEE/CVF volume 6, 2017.

5. Xu T, Feng ZH, Wu XJ, Kittler J. Learning Adaptive Discriminative Correlation Filters via Temporal Consistency Preserving Spatial Feature Selection for Robust Visual Object Tracking. IEEE Trans Image Process 2019; 28:5596-5609. https://doi.org/10.1109/TIP.2019.2919201

6. He K, Zhang X, Ren S, Sun J. “Deep residual learning for image recognition” inProceedings 265 of the IEEE conference on computer vision and pattern recognition 2016, pages 770–778

**Supplementary Tables**

**Table S1** The tumor classification accuracy with the proposed noise-rectifying (NR) loss in the internal test set.

|  | $\boldsymbol{\alpha=0}$ **(CE)** | $\boldsymbol{\alpha=0}$**.01** | $\boldsymbol{\alpha=0}$**.05** | $\boldsymbol{\alpha=0}$**.10** | $\boldsymbol{\alpha=0}$**.20** |
| --- | --- | --- | --- | --- | --- |
| **Accuracy** | 0.9217 | 0.9336 | 0.9457 | 0.9480 | 0.9290 |

Here the results are conducted with different values of α. The NR with α=0 equals to the Cross-Entropy (CE) loss. All scores are the average of ten experiments.

**Table S2** Microvascular segmentation results in the internal test set.

|  | **MPA** | **MIoU** | **FWIoU** | **DICE** |
| --- | --- | --- | --- | --- |
| **Normal Patch** | 0.9659 | 0.9438 | 0.9667 | 0.9896 |
| **MVI Patch** | 0.9085 | 0.8619 | 0.9198 | 0.9746 |

The results on normal and MVI patches are shown individually. We choose various segmentation metrics to evaluate the performance including MPA, MIoU, FWIoU, and DICE. All results are the average of ten experiments. HCC, hepatocellular carcinoma; MVI, microvascular invasion.

**Table S3** The results of MVI cell classification in the internal test set.

| **MVI Cell**  **Classification** | **Precision** | **Recall** | **F1 Score** |
| --- | --- | --- | --- |
|  | 0.7126 | 0.9452 | 0.8126 |

We apply various metrics for the cell classification task including precision, recall, and F1 score. All results are the average of ten experiments. MVI, microvascular invasion.

**Table S4** The reasons for MVI false-negative of 15 WSI cases diagnosed by pathologists.

| **Patient** | **Age（years）** | **Gender** | **Tumor number** | **Tumor diameter（cm）** | **Tumor differentiation** | **Number of MVI** | **Pathologists with assistance of model/Number** | **Change in AJCC T-stage** | **Reasons for missed diagnosis of MVI** |
| --- | --- | --- | --- | --- | --- | --- | --- | --- | --- |
| 1 | 48 | Male | 1 | 10.4 | Ⅲ | 2 | Yes/2 | T1b🡪T2 | Few cancer cells |
| 2 | 73 | Male | 1 | 7.8 | Ⅱ | 3 | Yes/2 | T1b🡪T2 | Few cancer cells |
| 3 | 53 | Male | 1 | 7.5 | Ⅲ | 3 | Yes/2 | T1b🡪T2 | Few cancer cells |
| 4 | 58 | Male | 1 | 1.3 | Ⅲ | 1 | Yes/1 | No | Inflammatory cells interference |
| 5 | 56 | Male | 1 | 2.0 | Ⅳ | 2 | Yes/2 | No | Few cancer cells |
| 6 | 48 | Female | 1 | 5.5 | Ⅳ | 2 | Yes/1 | T1b🡪T2 | Erythrocyte interference |
| 7 | 58 | Male | 1 | 2.1 | Ⅲ | 1 | Yes/1 | T1b🡪T2 | Difficult identification from micro satellite nodules |
| 8 | 37 | Male | 2 | 5.0 | Ⅱ | 3 | Yes/2 | No | Few cancer cells |
| 9 | 67 | Female | 2 | 8.0 | Ⅱ | 3 | Yes/2 | No | Few cancer cells |
| 10 | 57 | Male | 1 | 2.4 | Ⅲ | 1 | Yes/1 | T1b🡪T2 | Inflammatory cells interference |
| 11 | 63 | Male | 1 | 7.5 | Ⅱ | 2 | Yes/2 | T1b🡪T2 | Few cancer cells |
| 12 | 65 | Female | 1 | 6.0 | Ⅲ | 2 | Yes/1 | T1b🡪T2 | Few cancer cells |
| 13 | 45 | Male | 1 | 8.6 | Ⅱ | 3 | Yes/2 | T1b🡪T2 | Few cancer cells |
| 14 | 45 | Male | 3 | 2.5 | Ⅱ | 2 | No | No | Artificial contamination |
| 15 | 67 | Female | 1 | 3.5 | Ⅲ | 3 | No | No | Artificial contamination |

AJCC, American Joint Committee on Cancer；MVI, microvascular invasion; WSI, whole-slide image.

**Supplementary Figures**

**
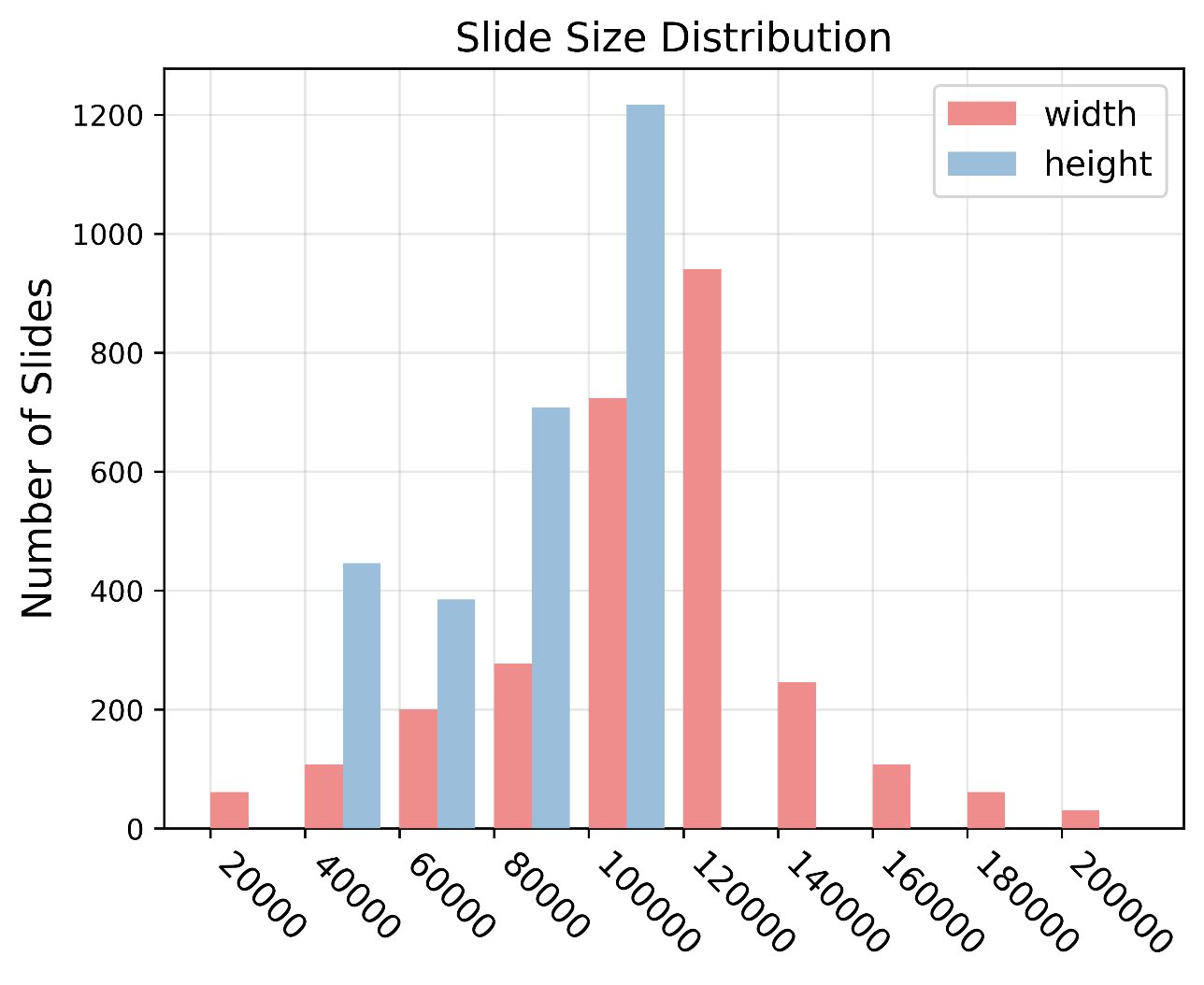
**

**Figure S1** Size distribution of the images widths (red) and heights (blue).


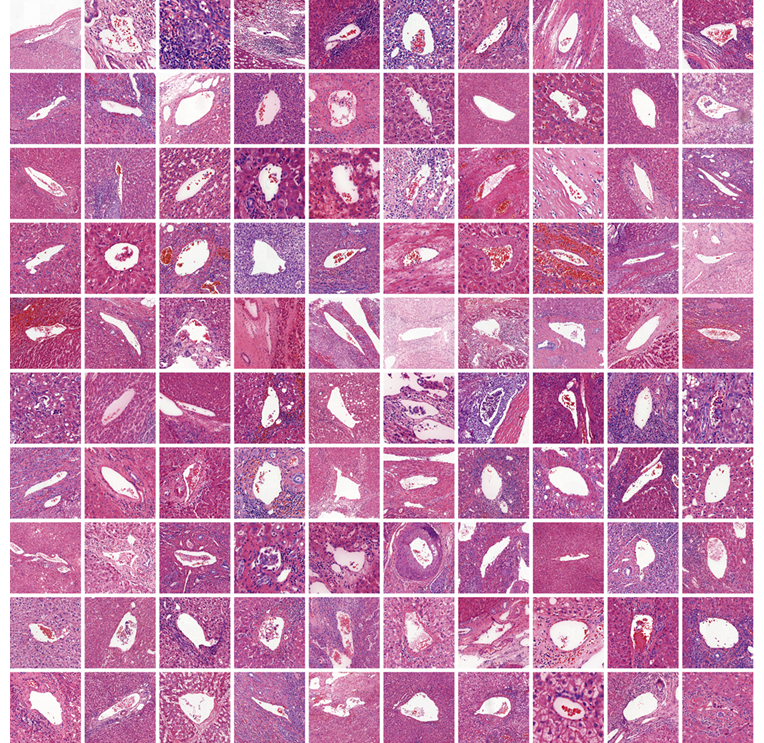


**Figure S2** Patches of microvascular segmentation in a WSI.
